# Anesthesia Strategies for the Perioperative Management of Severe Pulmonary Hypertension in Emergency Orthopedic Surgery: A Case Report

**DOI:** 10.7759/cureus.68836

**Published:** 2024-09-06

**Authors:** Ejaz Khan, Shoaib Nawaz, Kiran Inam, Malek Alali, Muhammad Waqas

**Affiliations:** 1 Anesthesiology, New York Medical College, Metropolitan Hospital Center, New York, USA; 2 Anesthesiology, Peshawar Institute of Cardiology, Peshawar, PAK

**Keywords:** anesthesia strategies, orthopedic surgery, perioperative care, pulmonary hypertension, spinal and epidural anesthesia

## Abstract

This case report details the perioperative challenges and anesthesia strategies in managing severe pulmonary hypertension (PH) during emergency orthopedic surgery. An 86-year-old male with multiple comorbidities, including severe PH, presented with a hip fracture. Multidisciplinary collaboration was crucial for preoperative optimization, including transfusions, antithrombotic discontinuation, and thromboprophylaxis initiation. Anesthesia management included the use of spinal anesthesia combined with a precautionary epidural catheter insertion, low-dose hyperbaric bupivacaine, and continuous monitoring to prevent hemodynamic instability. Postoperatively, the patient was closely monitored in the surgical intensive care unit. This case highlights the necessity of meticulous planning and proactive monitoring in optimizing outcomes for severe PH in emergency orthopedic surgery.

## Introduction

Pulmonary hypertension (PH) is defined as a condition characterized by elevated blood pressure in the pulmonary arteries, which increases the workload on the right side of the heart, potentially leading to right heart failure. PH presents a substantial challenge in the perioperative management of patients undergoing orthopedic surgeries, especially hip fractures. The severe impact of PH on pulmonary vascular resistance and right ventricular (RV) function dramatically increases the risk of perioperative cardiovascular collapse. Hip fractures are common among the elderly population, and given their associated morbidity and mortality risks, careful anesthesia management is crucial to ensure optimal patient outcomes.

Patients with PH are at an increased risk of postoperative mortality and serious morbidity, with rates ranging from 14% to 42% [1]. This includes acute RV failure, dysrhythmias, atrial fibrillation, coronary ischemia, renal failure, sepsis, stroke, and pulmonary complications [2]. Anesthesia strategies must prioritize the prevention of systemic hypotension and acute elevations in pulmonary arterial pressure to mitigate these risks effectively. Understanding the pathophysiological mechanisms underlying PH and right-sided heart failure is essential for effective perioperative management.

Patients with PH cannot accommodate alterations in RV preload or afterload induced by fluid shifts, medications, or changes in the autonomic nervous system, particularly under the stress of surgical intervention [3]. High airway pressures and excessive positive end-expiratory pressure (PEEP) should be avoided to optimize gas exchange and RV function. Enhancing RV contractility and pulmonary vasodilation may be necessary, though inotropes and systemic vasodilators should be used cautiously to prevent arrhythmias and systemic hypotension.

Despite the existence of general guidelines for managing PH during non-cardiac surgeries [4], there is a notable lack of detailed, case-specific strategies tailored to orthopedic procedures, particularly in patients with severe PH. While these strategies are well-established across various surgical contexts, our case report contributes to this field by providing specific insights into the perioperative management of a patient with severe PH undergoing emergency orthopedic surgery. We emphasize the importance of balancing effective pain management with the need to avoid exacerbation of PH, illustrating the practical application of these broader guidelines within an emergency orthopedic setting.

The patient has provided written consent for the publication of this article, and it complies with the relevant Enhancing the Quality and Transparency of Health Research (EQUATOR) guideline.

## Case presentation

An 86-year-old man with a medical history including hypertension, diabetes mellitus, and minimal nonobstructive coronary artery disease underwent percutaneous coronary intervention with stent placement in March 2023 while on Plavix (antiplatelet). He also had heart failure with a reduced ejection fraction (EF 30%), second-degree heart block with pacemaker insertion in 2017, atrial fibrillation managed with Eliquis (anticoagulant), chronic kidney disease, and altered mental status. The patient's condition was classified as WHO Group 2 PH (due to left heart disease) [4,5]. He presented to the emergency department after a fall, where he was found to be hemodynamically stable and alert, with a baseline mental status. Laboratory tests revealed a hemoglobin level of 8 g/dL, an international normalized ratio (INR) of 1.7, and an activated partial thromboplastin time (APTT) of 40 seconds. Imaging studies confirmed a left intertrochanteric femur fracture, with a normal head computed tomography (CT) scan. The patient was admitted following initial stabilization that included careful hemodynamic monitoring, administration of intravenous fluids, and oxygen supplementation to ensure adequate perfusion and oxygenation. The pain was managed with intravenous acetaminophen and a low dose of morphine.

**Figure 1:**
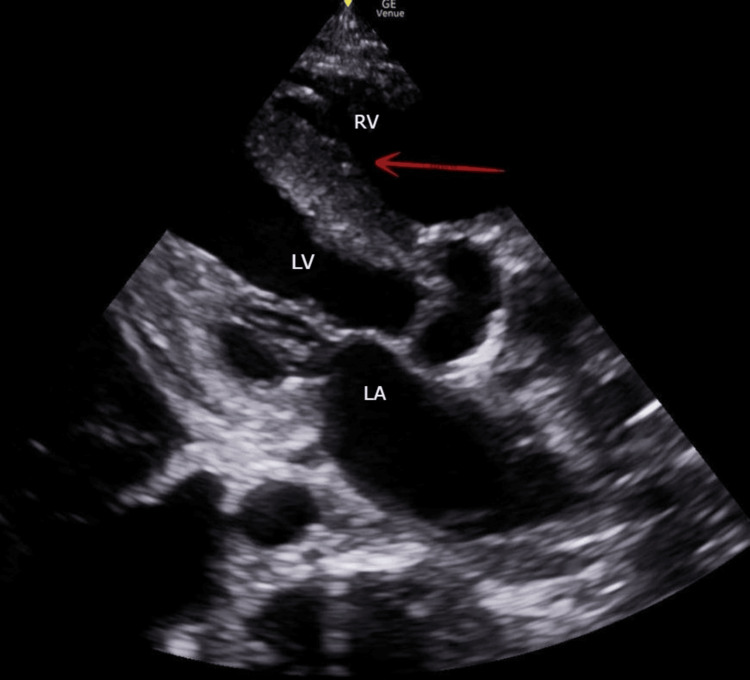
Deviation of the interventricular septum on a parasternal long-axis view (red arrow), and the heart chambers are labeled as RV, LV, and LA RV: right ventricle; LV: left ventricle; LA: left atrium

A recent echocardiogram demonstrated severe diffuse left ventricular hypokinesis with an EF of 20%, moderately dilated RV with increased wall thickness and hypokinesia, and severe tricuspid regurgitation. Echocardiographic findings also showed a deviation of the interventricular septum to the left as a sign of severe PH (see Figure 1).

**Figure 2:**
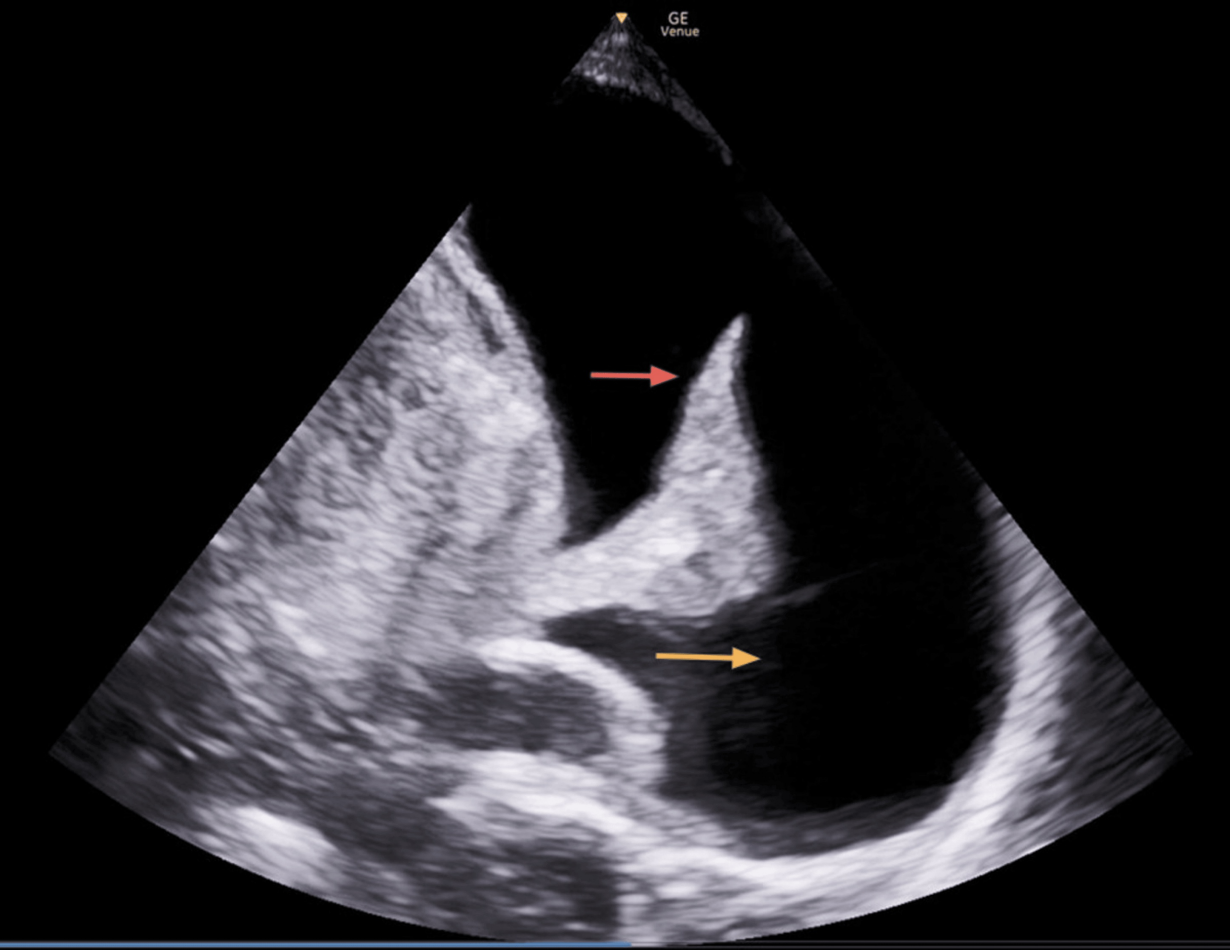
Left lung atelectasis (hyperechoic wedge indicated by red arrow) and large pulmonary effusion (hypoechoic area indicated by yellow arrow)

Point-of-care lung ultrasound showed a severely atelectatic left lung with a large pulmonary effusion (see Figure 2).

The patient was scheduled for intramedullary nail fixation of the left femur. The anesthesia team evaluated the patient and consulted with the orthopedic and internal medicine teams. Due to the complexity of the patient's medical condition, the surgery was delayed for further optimization, with a cardiology consultation obtained.

During this period, the patient received two units of packed red blood cells, resulting in an increase in hemoglobin to 10 g/dL. Additionally, antithrombotics (Plavix and Eliquis) were discontinued, and the prophylactic low-dose (40 mg) enoxaparin (low-molecular-weight heparin (LMWH)) was initiated which was stopped 12 hours before the scheduled surgery following neuraxial anesthesia guidelines [6]. On the day of the procedure, the patient was assessed in the preoperative area by the anesthesia team, and a bedside transthoracic echocardiogram was performed. A preoperative arterial line was inserted under ultrasound guidance to allow for continuous real-time monitoring of the patient's blood pressure and to facilitate frequent arterial blood gas analysis during the surgery.

In the operating room, intravenous fentanyl was administered for patient positioning, and low-dose (10 mg of bupivacaine) spinal anesthesia was performed, followed by the precautionary insertion of an epidural catheter. The patient remained hemodynamically stable throughout the procedure using a low-dose norepinephrine infusion to augment systemic vascular resistance. The surgery lasted approximately 1.5 hours, and the epidural catheter was not utilized and subsequently removed after the procedure.

Postoperatively, the patient was transferred to the surgical intensive care unit (SICU), where close monitoring was critical due to the high-risk nature of the case. Continuous monitoring included arterial blood pressure, central venous pressure, heart rate, oxygen saturation, and end-tidal CO2. Fluid balance was meticulously managed using a goal-directed approach, focusing on maintaining euvolemia while avoiding fluid overload to prevent the worsening of RV dysfunction. Crystalloids were administered accordingly, with careful monitoring of urine output and central venous pressure. Pain management was done with intravenous acetaminophen and low-dose morphine, without using epidural analgesia.

The patient was discharged from SICU care within 48 hours, meeting criteria that included hemodynamic stability without the need for vasopressor support, adequate pain control, and stable respiratory function. No complications were encountered in the immediate or early postoperative period. Upon discharge, the patient was given oral acetaminophen and advised to use a mild opioid for breakthrough pain if necessary.

## Discussion

The management of patients with PH undergoing surgery is a multifaceted process that begins before the operation and extends through the intraoperative and postoperative periods so that healthcare providers can minimize perioperative complications and enhance patient safety and well-being. Comprehensive preoperative assessment and optimization are essential to stabilize and optimize the patient's condition before surgery. This holistic approach to patient care involves a collaborative effort among various medical specialties, including anesthesia, surgery, internal medicine, and cardiology, to address the complex medical needs of these patients effectively.

Literature review reveals that underlying PH significantly worsens perioperative outcomes, beyond traditional risk factors like coronary artery disease, diabetes, congestive heart failure, chronic renal insufficiency, and the American Society of Anesthesiologists (ASA) class. Patients with PH experience higher rates of congestive heart failure, respiratory failure, and hemodynamic instability, longer ICU stays, and increased 30-day readmission rates compared to those without PH [7]. Additionally, PH can significantly affect the ASA physical status classification. In this case report, the patient with severe PH was classified as ASA Class 4 which underscores the complexity of managing such patients and the need for vigilant monitoring and optimized anesthetic and surgical strategies to mitigate perioperative risks.

In contrast to prior studies that emphasize broad perioperative management guidelines, our case illustrates the benefits of personalized care tailored to the unique needs of patients with severe PH. For our patient, the collaborative effort among different specialties was imperative to optimize the patient's condition effectively. Although the surgery was initially deemed an emergency, a five-day delay was necessary to optimize the patient's condition as recommended by the cardiology team. Despite this delay, the patient remained inadequately optimized and ultimately underwent the procedure under emergency protocols.

In the future when managing anesthesia for patients with severe PH, meticulous planning is crucial to mitigate perioperative risks. Preoperative evaluations by an anesthesiologist and a PH specialist should be conducted whenever possible. In emergencies, consulting a PH specialist before surgery may not be feasible; therefore, an experienced anesthesiologist should lead perioperative care. Diagnostic tools may include a formal transthoracic echocardiogram by a cardiac sonographer or a point-of-care cardiac ultrasound by a qualified clinician to assess right-sided heart function for immediate risk stratification. For elective surgeries, a multidisciplinary discussion on the urgency and necessity of the operation should precede surgical planning.

In this case, a spinal anesthesia technique was selected to provide intraoperative anesthesia while minimizing systemic effects. A low dose of hyperbaric bupivacaine was used to achieve effective pain control without causing excessive vasodilation, which can reduce systemic vascular resistance, worsening PH by increasing RV workload and potentially leading to right heart failure. Given the patient's high-risk status, an epidural catheter was inserted as a precautionary measure to allow for additional intraoperative and postoperative analgesia if needed. However, the patient's pain was effectively managed with low-dose spinal anesthesia, rendering the use of the epidural catheter unnecessary. Additionally, the preoperative insertion of an arterial line under ultrasound guidance facilitated the real-time monitoring of hemodynamic parameters during induction and throughout the surgical procedure, allowing for prompt intervention in case of hemodynamic instability. 

During the surgery, the use of low-dose norepinephrine infusion was instrumental in improving perfusion to the right coronary artery, reducing the pulmonary/systemic vascular resistance ratio, and enhancing RV performance, thereby marginally improving cardiac output [4]. Continuous vigilance and close monitoring of vital signs, arterial blood gases, and central venous pressure were maintained to promptly detect and manage any hemodynamic disturbances, including hypercarbia, which can worsen PH by causing vasoconstriction and increasing pulmonary artery pressure.

Postoperatively, the patient remained in the SICU for two days under continuous observation. Pain management included intravenous acetaminophen and low-dose morphine for breakthrough pain, thereby mitigating the risk of vasodilation-induced hypotension associated with epidural analgesia. The decision to avoid using an epidural catheter was particularly crucial given the patient's susceptibility to further hypotension, as the patient had required a norepinephrine infusion, which was successfully discontinued after several hours. On discharge, the patient was prescribed oral acetaminophen, with a mild opioid for breakthrough pain if needed. This strategy was important for optimizing RV function and preventing pulmonary hypertensive crises.

This comprehensive approach to anesthesia management in patients with severe PH underscores the importance of meticulous planning, proactive monitoring, and multidisciplinary collaboration to optimize patient outcomes and minimize perioperative complications. However, this case report has limitations. The findings are based on a single patient, which may not be generalizable to all cases involving severe PH. Variability in individual patient responses and the specific context of emergency orthopedic procedures may limit the broader applicability of these strategies. Future research should address these limitations by evaluating a larger cohort and exploring the effectiveness of these approaches in different surgical settings.

## Conclusions

This case highlights the complexities of managing patients with severe PH undergoing emergency orthopedic surgery. A multidisciplinary approach, careful anesthesia planning, and vigilant intraoperative and postoperative management are essential to optimize outcomes and minimize complications. This report underscores the importance of tailoring anesthesia strategies to the unique challenges presented by PH, ensuring a balanced approach to both pain management and hemodynamic stability.
